# Long-Term Kinetics of SARS-CoV-2 Antibodies and Impact of Inactivated Vaccine on SARS-CoV-2 Antibodies Based on a COVID-19 Patients Cohort

**DOI:** 10.3389/fimmu.2022.829665

**Published:** 2022-01-27

**Authors:** Shihan Zhang, Ke Xu, Chuchu Li, Lu Zhou, Xiaoxiao Kong, Jiefu Peng, Fengcai Zhu, Changjun Bao, Hui Jin, Qiang Gao, Xing Zhao, Liguo Zhu

**Affiliations:** ^1^ Department of Epidemiology and Health Statistics, School of Public Health, Southeast University, Nanjing, China; ^2^ Key Laboratory of Environmental Medicine Engineering, Ministry of Education, School of Public Health, Southeast University, Nanjing, China; ^3^ Department of Acute Infectious Disease Control and Prevention, Jiangsu Provincial Center for Disease Control and Prevention, Nanjing, China; ^4^ National Health Commission (NHC) Key Laboratory of Enteric Pathogenic Microbiology, Jiangsu Provincial Center for Disease Control and Prevention, Nanjing, China; ^5^ Key Laboratory of Infectious Diseases, School of Public Health, Nanjing Medical University, Nanjing, China; ^6^ Department of Acute Infectious Disease Control and Prevention, Huai’an Center for Disease Control and Prevention, Huaian, China; ^7^ Department of Acute Infectious Disease Control and Prevention, Lianyungang Center for Disease Control and Prevention, Lianyungang, China; ^8^ Jiangsu Key Lab of Cancer Biomarkers, Prevention and Treatment, Jiangsu Collaborative Innovation Center for Cancer Medicine, Nanjing Medical University, Nanjing, China

**Keywords:** SARS-CoV-2, antibody responses, natural infection, vaccination, long-term kinetics

## Abstract

**Background:**

Understanding the long-term kinetic characteristics of SARS-CoV-2 antibodies and the impact of inactivated vaccines on SARS-CoV-2 antibodies in convalescent patients can provide information for developing and improving vaccination strategies in such populations.

**Methods:**

In this cohort, 402 convalescent patients who tested positive for SARS-CoV-2 by RT-PCR from 1 January to 22 June 2020 in Jiangsu, China, were enrolled. The epidemiological data included demographics, symptom onset, and vaccination history. Blood samples were collected and tested for antibody levels of specific IgG, IgM, RBD-IgG, S-IgG, and neutralizing antibodies using a the commercial magnetic chemiluminescence enzyme immunoassay.

**Results:**

The median follow-up time after symptom onset was 15.6 months (IQR, 14.6 to 15.8). Of the 402 convalescent patients, 44 (13.84%) received an inactivated vaccine against COVID-19. A total of 255 (80.19%) patients were IgG-positive and 65 (20.44%) were IgM-positive. The neutralizing antibody was 83.02%. Compared with non-vaccinated individuals, the IgG antibody levels in vaccinated people were higher (P=0.007). Similarly, antibody levels for RBD-IgG, S-IgG, and neutralizing antibodies were all highly increased in vaccinated individuals (P<0.05). IgG levels were significantly higher after vaccination than before vaccination in the same population. IgG levels in those who received ‘single dose and ≥14d’ were similar to those with two doses (P>0.05). Similar conclusions were drawn for RBD-IgG and the neutralizing antibody.

**Conclusion:**

15.6 months after symptom onset, the majority of participants remained positive for serum-specific IgG, RBD-IgG, S-IgG, and neutralizing antibodies. For convalescent patients, a single dose of inactivated vaccine against COVID-19 can further boost antibody titres.

## Introduction

Coronavirus disease 2019 (COVID-19) is an infectious disease caused by the novel severe acute respiratory syndrome coronavirus 2 (SARS-CoV-2). It was first discovered in December 2019, in the city of Wuhan, China, and subsequently spread to countries around the world, causing a pandemic. As of 15 October 2021, there have been 239,437,517 confirmed cases, including 4,879,235 deaths, reported to the WHO (1).

Based on serological studies of naturally infected populations, IgM antibodies are the first to be expressed and are mainly present in the circulation, promoting antigenic modulation (2, 3). IgG antibodies begin to appear later in the immune response because they undergo affinity maturation through somatic mutations, resulting in high affinity for the target antigen and an enhanced ability to neutralise the pathogen (2). SARS-CoV-2 particles contain four main structural proteins: spike protein (S), envelope protein (E), membrane protein (M), and nucleocapsid protein (N). Of these, spike protein (S) interacts with hosts’ ACE2 and TMPRSS2 receptors for entry (4). Therefore, in terms of protection, the S protein is considered to be the most relevant antigen causing key antibody responses (5). Neutralizing antibodies play an important role in the prevention and vaccine development of COVID-19 (6). Currently, there is still a large global population in various forms of temporary quarantine to limit the spread of the virus, resulting in severe disruptions to international travel and local socioeconomic activities (2). Therefore, there is an urgent need to better understand the nature and duration of immunization against SARS-CoV-2 since almost all epidemiological models, vaccination campaigns, and public health measures assume some degree of immunity during the COVID-19 recovery period (7–9). Some studies in Iceland and the United States have shown that antibodies persist for more than 4 months after infection, but other studies have reported rapid fading of antibodies within 3-4 months (10). Most of the available studies have explored the outcome of specific antibodies at 6 months or even 1 year. Nevertheless, we urgently need to understand the long-term durability of SARS-CoV-2-specific IgG and IgM and neutralizing antibody (nAb) responses after symptom onset or RT-PCR confirmation, which is critical for learning the characteristics or patterns of antibody depletion. In addition, the increasing number of COVID-19 convalescent patients will want to know if they still need the vaccine and how many doses are sufficient.

In the first round of follow-up of 284 convalescent patients between August and October 2020, we obtained short-term characteristics of the dynamic changes in antibodies (3). In the current study, we continued a second round of follow-up of 318 patients recovering from COVID-19 from December 2020 to June 2021, in addition to testing for specific antibodies IgG, IgM, and neutralizing antibodies, we also measured antibody levels for IgG subtypes and collected information on vaccination in these subjects. The aim was to understand the dynamic characteristics and duration of specific antibodies in the naturally infected population over a longer period of time, and the effect of vaccination on antibody levels in all aspects.

## Methods

### Patients and Data Collection

**Figure 1:**
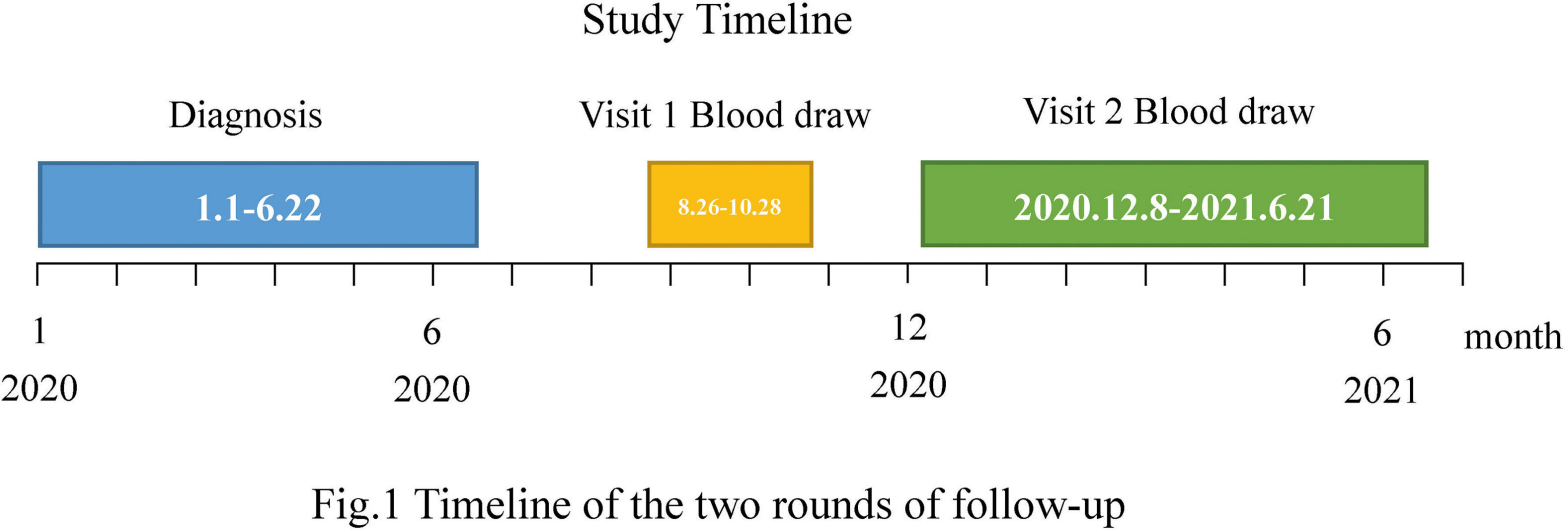
Timeline of the two rounds of follow-up. A total of 402 COVID-19-cured patients (confirmed from January to June 2020) participated in the follow-up visits. Of these, 284 underwent the first round of follow-up (blood draw period from 26 August to 28 October 2020) and 318 participated in the second round of follow-up (blood draw period from 8 December 2020 to 21 June 2021).

A total of 402 COVID-19-cured patients (confirmed from January to June 2020) in Jiangsu Province, China, were followed up in two rounds between 26 August 2020 and 28 October 2020 (the first round) and between 8 December 2020 and 21 June 2021 (the second round) (**Figure 1**). Of these, 284 patients participated in the first follow-up visit, 318 in the second, and 228 in both rounds. According to the Diagnosis and Treatment Protocol for Novel Coronavirus Pneumonia released by the National Health Commission of the People’s Republic of China, the detection of novel coronavirus nucleic acids in specimens such as nasal and oropharyngeal swabs, sputum and other lower respiratory secretions, blood, stool, and urine using real-time reverse transcriptase-polymerase chain reaction (RT-PCR) and (next-generation sequencing) (NGS) were the primary criteria for diagnosis. All subjects included in this cohort met the above criteria. As in the first round (3), blood samples were collected from the second round of follow-up to measure the level of specific antibodies against SARS-CoV-2 and to monitor the dynamics of the antibodies in the body. The clinical data included immunization history of inactivated vaccine against COVID-19, demographics, and acute phase disease severity classification (Version 8 Diagnosis and Treatment Protocol for COVID-19).

### CLIA-Based (Chemiluminescence Immunoassay) Detection of Specific Antibodies and Neutralizing Antibodies Against SARS-CoV-2

In the second round of follow-up, participants’ serum samples were used to test the levels of the following antibodies: specific IgG, IgM, IgG subtypes (RBD-IgG, S-IgG, and N-IgG), and neutralizing antibodies. These specific antibodies were tested using the following commercial kits: Novel Coronavirus (2019-nCoV) IgM/IgG antibody diagnostic kit (plate CLIA) supplied by Bioscience Co. (China National Medical Products Administration, approval numbers 20203400183 [IgG] and 20203400182 [IgM]) on an automated magnetic chemiluminescence analyzer (Axceed 260; Bioscience). Detailed information on the principle of the test, the procedure, and the sensitivity and specificity of this kit can be found in the first round of follow-up (3). The levels of neutralizing antibodies against SARS-CoV-2 were calculated using ACE2-RBD inhibiting antibody concentration using the research kit (Bioscience Co.) conducted on the same automated magnetic chemiluminescence analyser. The chemiluminescent signal was measured as a relative light unit (RLU) using the optical system of the analyser. The titres of SARS-CoV-2 ACE2 competitive antibodies in the serum sample were measured as S/CO (sample/cut-off) by comparing the RLU of a sample to the cut-off determined from standard curves. If the S/CO value is ≥2.0, the test result is positive, while if the S/CO value is <1.0, the result is considered negative; if between 1.0-2.0, the judgment is indeterminate and comprehensive judgment is recommended. The detection performance of the MCLIA kits for neutralization antibodies was reported by the manufacturer and the coincidence rate was 85-90%.

### Statistical Analyses

Continuous variables were expressed as means ± 95%CI, and significance was calculated using the two-tailed t-test, one-way ANOVA or Wilcoxon rank sum (Mann–Whitney) test as appropriate, and categorical variables were presented as percentages, and significance was calculated using the chi-square test or Fisher’s exact test as appropriate. The change in IgG positive rates over the follow-up period was presented as line plots and the change in the level of IgG quantification was shown as bar plots. Evaluation of the effectiveness of vaccination doses was shown on a violin plot. Multivariate regression analyses of antibody positive rates and levels with factors, such as sex, age, disease severity, and vaccination were performed to confirm the cross-effect between these factors. Stata (version 15.0) and GraphPad Prism (version 9.0) software were used for the statistical analysis. Statistical significance was considered at P<0.05 (ns: no significance; *p<0.05; **p<0.01; ***p<0.001; ****p<0.0001).

## Results

### Demographic and Clinical Characteristics of the Subjects in the Follow-Up

A total of 318 COVID-19 convalescent patients participated in this round of follow-up from December 2020 to June 2021, with a median follow-up duration of 15.6 months (inter-quartile range [IQR], 14.6 to 15.8) and a maximum duration of 18 months. The demographic and clinical characteristics of the subjects are presented in **Table 1**. The female to male ratio was 52.8% vs. 47.2%. The age range varied from 8 to 91 years and was concentrated in the 30-39 years (24.84%) and 50-59 years (23.27%) age groups. The mean age of all patients was 45.21 years (IQR, 33-57). Depending on the severity of disease, the normal type accounted for the largest proportion (50.94%), followed by the asymptomatic type (28.93%). In these convalescent patients, 44(13.84%) received an inactivated vaccine against COVID-19. There were no statistically significant differences in the demographic and clinical characteristics between the vaccinated and unvaccinated groups.

**Table 1:** Demographic and clinical characteristics of the subjects in this round of follow-up.

Characteristics	All patients	Unvaccinated	Vaccinated	P^a^
Total	318	274	44	
Gender				0.222
Male	150(47.2)	133(48.54)	17(38.64)	
Female	168(52.8)	141(51.46)	27(61.36)	
Age (mean,95%CI)	45.21 (43.44,46.98)	45.46 (43.49,47.42)	43.70 (40.00,47.40)	0.503
Age (ys)				0.201
<20	17(5.35)	17(6.20)	0(0.00)	
20~	32(10.06)	28(10.22)	4(9.09)	
30~	79(24.84)	64(23.36)	15(34.09)	
40~	54(16.98)	44(16.06)	10(22.73)	
50~	74(23.27)	64(23.36)	10(22.73)	
≥60	62(19.50)	57(20.80)	5(11.36)	
Severity of disease				0.100
Asymptomatic type	92(28.93)	82(29.93)	10(22.73)	
Mild type	60(18.87)	46(16.79)	14(31.82)	
Normal type	162(50.94)	143(52.19)	19(43.18)	
Severe/Critical type	4(1.26)	3(1.09)	1(2.27)	
SARS-CoV-2 vaccination				
Unvaccinated	274(86.16)			
Vaccinated	44(13.84)			

^a^Chi-square test or fisher’s exact test as appropriate;

P < 0.05 represents significant difference.

### Distribution of IgM/IgG Positive Rates and Antibody Levels

As shown in **Table 2**, among the 318 individuals who participated in this round of follow-up, 255 (80.19%) were IgG-positive, and 65 (20.44%) were IgM-positive. The antibody level for IgG was 9.73 (95%CI, 8.28-11.19) in those patients with positive IgG, meanwhile, the antibody level for IgM was 4.85 (95%CI, 2.85-6.86) in those patients with positive IgM. Compared with unvaccinated individuals, the IgG antibody levels of vaccinated people were higher (*P*=0.007). However, there was no statistically significant difference in IgM antibody levels between those with and without a history of vaccination. **Table 2** and S1 illustrate the correlation of IgG positive rates with age and severity of disease in all convalescent patients and unvaccinated convalescent patients (*P*<0.05). **Table S2** suggests that the rate of IgG positivity in vaccinated convalescent individuals was related to sex and disease severity (*P*<0.05). The IgG positive rate was significantly greater in females than in males (96.30% vs. 76.47%), and there was a positive correlation with disease severity. However, we did not observe an association between IgG levels, sex, and disease severity in the vaccinated population (*P*>0.05).

**Table 2:** Distribution of IgM/IgG among overall convalescent patients.

	IgG positive number (%)	P	IgG antibody levels (mean,95%CI)	P	IgM positive number (%)	P	IgM antibody levels (mean,95%CI)	P
Overall	255(80.19)		9.73(8.28,11.19)		65(20.44)		4.85(2.85,6.86)	
Gender		0.227		0.520		0.644		0.564
Male	116(77.33)		9.21(6.99,11.44)		29(19.33)		4.20(1.79,6.61)	
Female	139(82.74)		10.17(8.24,12.10)		36(21.43)		5.38(2.21,8.55)	
Age (ys)		0.010^*^		0.280		0.401		0.082
<20	9(52.94)		4.70(2.57,6.82)		1(5.88)		1.44	
20~	23(71.88)		10.60(3.44,17.77)		8(25.00)		2.56(1.17,3.95)	
30~	61(77.22)		7.54(5.84,9.24)		17(21.52)		3.47(1.85,5.09)	
40~	45(83.33)		10.71(7.81,13.61)		15(27.78)		3.74(1.87,5.61)	
50~	67(90.54)		11.77(7.98,15.56)		13(17.57)		11.02(2.06,19.97)	
≥60	50(80.65)		9.31(6.75,11.86)		11(17.74)		3.21(1.81,4.61)	
Severity of disease		0.003^**^		0.330		0.489		0.685
Asymptomatic type	62(67.39)		7.33(5.39,9.27)		17(18.48)		3.90(1.93,5.88)	
Mild type	50(83.33)		10.26(7.72,12.80)		13(21.67)		3.64(2.12,5.16)	
Normal type	139(85.80)		10.60(8.27,12.93)		33(20.37)		6.02(2.26,9.79)	
Severe/Critical type	4(100.0)		10.37(2.93,17.82)		2(50.0)		1.61(0.92,2.31)	
SARS-CoV-2 vaccination		0.130		0.007^**^		0.226		0.801
Unvaccinated	216(78.83)		8.90(7.32,10.48)		53(19.34)		4.98(2.55,7.40)	
Vaccinated	39(88.64)		14.37(11.02,17.72)		12(27.27)		4.31(2.17,6.46)	

P < 0.05 represents significant difference. *p < 0.05; **p < 0.01.

Comparisons of IgM/IgG positive rates and antibody levels between convalescent patients with and without vaccination are shown in the Supplemental material (**Tables S3–S6**). The rate of IgG positivity was only slightly higher in the vaccinated women (*P*=0.042). However, IgG antibody levels were elevated in overall, male, 30-39 years age group, and asymptomatic and mild type subjects(*P*<0.05). Relative to the unvaccinated group, the IgM positive rate in the vaccinated group was only increased in the mild type, and IgM antibody levels were only increased in the 30-39 age group (*P*=0.028 and *P*=0.003, respectively).

### Distribution of IgM/IgG Combinations in Second-Round Follow-Up Subjects

15.6 months after symptom onset, the predominance of IgG single-positive was 62.58% at the end of the second follow-up. The percentage of IgM single-positive was 2.83%, IgM/IgG double-positive was 17.61% and IgM/IgG double-negative was 16.98%. The proportion of IgG single-positive and IgM/IgG double-positive cases increased as the severity of the acute infection worsened (*P*<0.001). However, there was no difference in this distribution with or without a history of vaccination (*P*>0.05). The relevant data are shown in **Table 3**.

**Table 3:** Distribution of IgM/IgG combinations in second-round follow-up subjects.

	IgM/IgG double-negative	IgM single-positive	IgG single-positive	IgM/IgG double-positive	P
Overall	54(16.98)	9(2.83)	199(62.58)	56(17.61)	
Gender					0.644
male	29(19.33)	5(3.33)	92(61.33)	24(16.00)	
female	25(14.88)	4(2.38)	107(63.69)	32(19.05)	
Age (ys)					0.044^*^
<20	8(47.06)	0(0)	8(47.06)	1(5.88)	
20~	8(25.00)	1(3.13)	16(50.00)	7(21.88)	
30~	14(17.72)	4(5.06)	48(60.76)	13(16.46)	
40~	8(14.81)	1(1.85)	31(57.41)	14(25.93)	
50~	5(6.76)	2(2.7)	56(75.68)	11(14.86)	
≥60	11(17.74)	1(1.61)	40(64.52)	10(16.13)	
Severity of disease					<0.001^***^
Asymptomatic type	28(30.43)	2(2.17)	47(51.09)	15(16.4)	
Mild type	8(13.33)	2(3.33)	39(65.00)	11(18.34)	
Normal type	18(11.11)	5(3.09)	111(68.52)	28(17.28)	
Severe/Critical type	0(0)	0(0)	4(50.00)	4(50.00)	
SARS-CoV-2 vaccination					0.318
Unvaccinated	50(18.25)	8(2.92)	171(62.41)	45(16.42)	
Vaccinated	4(9.09)	1(2.27)	28(63.64)	11(25.00)	

P < 0.05 represents significant difference. *p < 0.05; ***p < 0.001.

### Distribution of Positive Rates and Antibody Levels for IgG Subtypes and Neutralizing Antibodies

At the second follow-up, the positive rate of RBD-IgG was 89.31%, S-IgG was 91.51%, neutralizing antibody was 83.02%, and N-IgG was 78.93%. We found that the positive rates of IgG subtypes and neutralizing antibodies correlated with disease severity (*P*<0.05) and showed a positive correlation. No differences were found in the positive rates of IgG subtypes and neutralizing antibodies between those with and without vaccination (*P*>0.05). In terms of antibody levels, the mean for RBD-IgG was 11.37 (95% CI, 10.00-12.74), S-IgG was 15.27 (95% CI, 13.46-17.08), and neutralizing antibodies reached 18.13 (95% CI, 16.31-19.96). Significantly, antibody levels for RBD-IgG, S-IgG, N-IgG, and neutralizing antibodies were all highly increased in vaccinated individuals relative to unvaccinated individuals (*P*<0.05). The results are shown in **Table 4**.

**Table 4:** Distribution of positive rates and antibody levels for IgG subtypes and neutralizing antibodies.

	RBD-IgG positive number(%)	P	S-IgG positive number(%)	P	N-IgG positive number(%)	P	Nab positive number(%)	P	RBD-IgG antibody levels (mean,95%CI)	P	S-IgG antibody levels (mean,95%CI)	P	N-IgG antibody levels (mean,95%CI)	P	Nab antibody levels (mean,95%CI)	P
Overall	284(89.31)		291(91.51)		251(78.93)		264(83.02)		11.37(10.00,12.74)		15.27(13.46,17.08)		9.02(7.54,10.50)		18.13(16.31,19.96)	
Gender		0.150		0.188		0.701		0.449		0.677		0.530		0.901		0.247
Male	130(86.67)		134(89.33)		117(78.00)		122(81.33)		11.05(8.85,13.25)		14.65(11.78,17.51)		9.12(6.80,11.44)		16.98(14.36,19.59)	
Female	154(91.67)		157(93.45)		134(79.76)		142(84.52)		11.64(9.89,13.38)		15.80(13.49,18.12)		8.93(7.00,10.85)		19.13(16.57,21.69)	
Age (ys)		0.603		0.422		<0.001^***^		0.505		0.137		0.088		0.005^**^		0.708
<20	15(88.24)		15(88.24)		7(41.48)		14(82.35)		5.85(2.57,9.12)		8.26(3.56,12.96)		3.39(1.70,5.07)		12.67(4.61,20.72)	
20~	28(87.50)		28(87.50)		22(68.75)		26(81.25)		11.48(5.34,17.62)		14.42(7.31,21.52)		5.56(2.92,8.20)		16.33(9.96,22.70)	
30~	67(84.81)		69(87.34)		59(74.68)		62(78.48)		9.83(7.61,12.05)		12.58(9.80,15.36)		5.60(4.33,6.87)		18.83(14.95,22.71)	
40~	50(92.59)		50(92.59)		47(87.04)		44(81.48)		11.05(8.29,13.80)		15.42(11.29,19.56)		9.54(6.59,12.49)		19.26(15.01,23.50)	
50~	69(93.24)		71(95.95)		63(85.14)		67(90.54)		14.15(10.52,17.77)		19.13(14.50,23.77)		13.22(9.01,17.44)		19.10(15.29,22.91)	
≥60	55(88.71)		58(93.55)		53(85.48)		51(82.26)		11.51(8.85,14.16)		15.84(11.97,19.71)		9.53(5.89,13.17)		17.47(13.22,21.72)	
Severity of disease		0.003^**^		0.013^*^		<0.001^***^		0.004^**^		0.184		0.116		0.349		0.106
Asymptomatic type	73(79.35)		77(83.70)		57(61.96)		66(71.74)		8.76(6.68,10.84)		11.65(8.84,14.46)		7.45(5.11,9.80)		14.41(11.16,17.67)	
Mild type	57(95.00)		58(96.67)		54(90.00)		55(91.67)		12.33(9.81,14.85)		17.15(13.74,20.56)		10.94(7.14,14.74)		19.37(15.05,23.69)	
Normal type	150(92.59)		152(93.83)		136(83.95)		139(85.80)		12.26(10.06,14.46)		16.27(13.41,19.13)		8.75(6.69,10.81)		19.20(16.64,21.76)	
Severe/Critical type	4(100.00)		4(100.00)		4(100.00)		4(100.00)		12.04(6.61,17.47)		19.70(3.52,35.88)		14.48(-5.26,34.22)		25.64(-0.51,51.79)	
SARS-CoV-2 vaccination		0.711		0.878		0.193		0.285		0.003^**^		0.0001^***^		<0.001^***^		<0.001^***^
Unvaccinated	244(89.05)		251(91.61)		213(77.74)		225(82.12)		10.54(9.06,12.02)		13.86(11.99,15.74)		7.33(6.03,8.62)		16.11(14.21,18.01)	
Vaccinated	40(90.91)		40(90.91)		38(86.36)		39(88.64)		16.41(12.97,19.85)		24.11(18.81,29.40)		18.50(12.60,24.40)		29.83(25.62,34.04)	
Months after symptom onset		0.029^*^		0.144		0.014^*^		0.002^**^		0.104		0.074		0.850		0.0007^***^
9~10M	8(100.00)		8(100.00)		8(100.00)		8(100.00)		5.43(1.46,9.40)		8.55(2.56,14.54)		5.82(1.33,10.32)		13.83(1.97,25.69)	
11~12M	7(63.64)		8(72.73)		5(45.45)		6(54.55)		17.91(0.28,35.54)		21.82(0.02,43.62)		10.46(0.37,20.55)		13.69(-0.95,28.34)	
13~14M	64(86.49)		67(90.54)		57(77.03)		54(72.97)		9.05(6.92,11.18)		11.89(9.20,14.58)		8.18(5.43,10.93)		12.82(9.28,16.36)	
15~16M	195(90.70)		198(92.09)		171(79.53)		186(86.51)		12.02(10.25,13.80)		16.07(13.74,18.40)		9.27(7.33,11.20)		19.20(17.00,21.39)	
17~18M	10(100.00)		10(100.00)		10(100.00)		10(100.00)		13.62(8.60,18.64)		22.24(13.20,31.28)		11.34(4.13,18.54)		33.21(23.92,42.50)	

P < 0.05 represents significant difference. *p <0.05; **p<0.01; ***p<0.001; ****p<0.0001.

**Figure 2:**
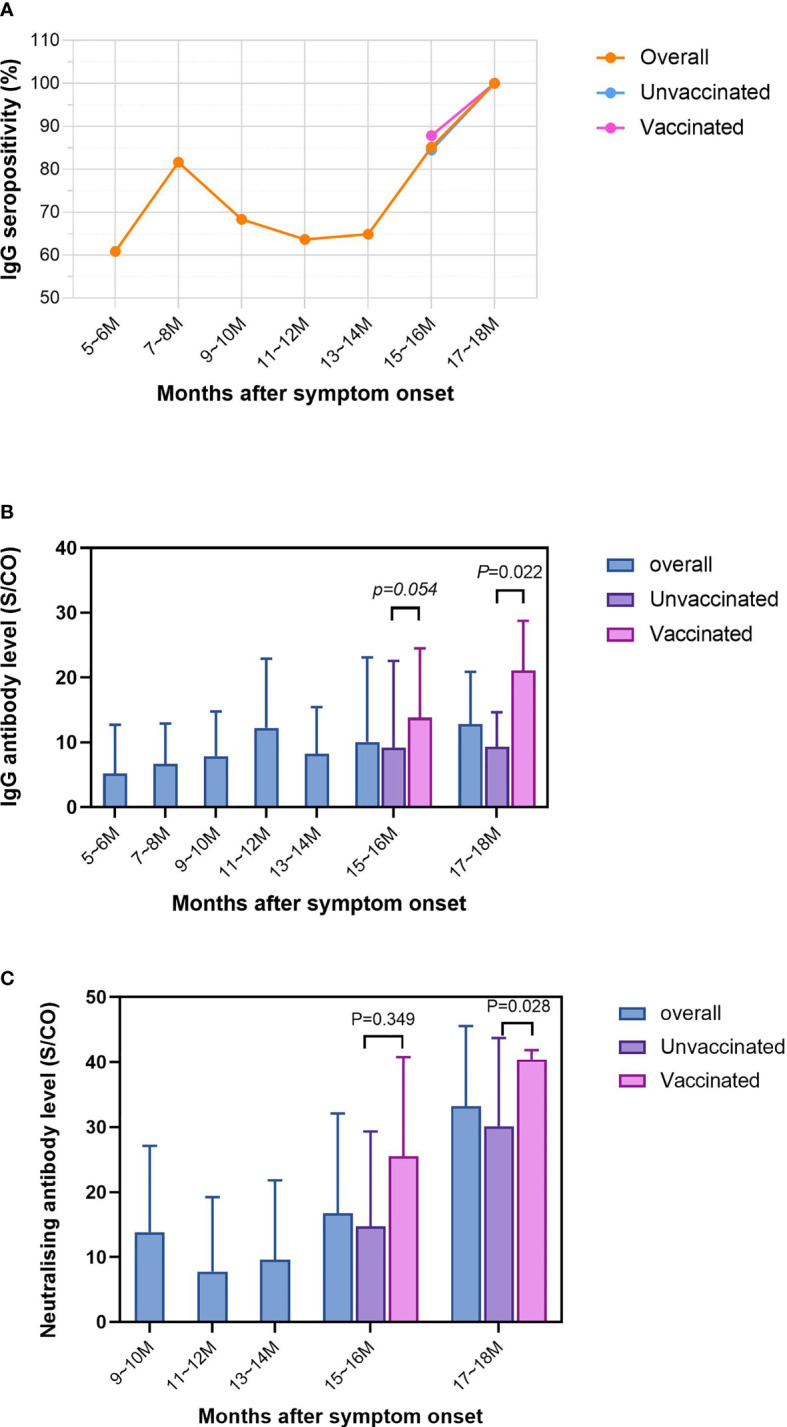
Dynamics of the IgG positive rate and antibody levels during the two follow-up rounds. The length of follow-up ranged from 5.4 months to 17.4 months. At two-month intervals, the horizontal axis has been divided into seven equal parts, 5~6 months, 7~8 months, 9~10 months, 11~12 months, 13~14 months, 15~16 months and 17~18 months. **(A)** Trends in IgG positive rate over months after symptom onset in overall, unvaccinated and vaccinated convalescent patients. **(B)** Trends in IgG antibody levels over months after symptom onset in overall, unvaccinated and vaccinated convalescent patients. **(C)** Trends in neutralising antibody levels over months after symptom onset in overall, unvaccinated and vaccinated convalescent patients. P values were determined applying a two-tailed Mann-Whitney U test. P<0.05 was considered to be statistically significant.

Comparisons of levels for IgG subtypes and neutralizing antibodies between convalescent patients with and without vaccination are shown in the Supplemental material (**Tables S9–S12**). Remarkably, compared to the unvaccinated group, the level of RBD-IgG antibodies in vaccinees was significantly higher in males, 40-49 age group and patients with mild disease (*P*<0.05). For S-IgG, antibody levels were significantly higher in vaccinated individuals than in unvaccinated individuals, regardless of sex. Similar conclusions could be drawn for the 30-39 years and 40-49 years age groups and convalescent patients with mild and normal type disease (*P*<0.05). For neutralizing antibodies, we found that antibody levels were significantly higher in vaccinated individuals than in unvaccinated individuals, irrespective of sex and disease severity (*P*<0.05) (**Figure 2C**). In addition, similar findings were found in the 30-39, 50-59, and ≥60 age groups.

### Kinetics of the IgG Positive Rates and Antibody Levels During the Two Follow-up Rounds

Of the 402 patients that participated in the follow-up, the length of follow-up ranged from 5.4 months to 17.4 months. The horizontal axis was divided into seven equal parts: 5-6 months, 7-8 months, 9-10 months, 11-12 months, 13-14 months, 15-16 months and 17-18 months. The variation in the total positive rate, the positive rate in the unvaccinated group and the positive rate in the vaccinated group are shown in **Figure 2A**. There was an overall upward trend in the IgG positive rate over the months after symptom onset. The lowest positive rate of 60.87% was recorded at 5-6 months, reaching a maximum of 100% in 17-18 months. Interestingly, there was a sudden increase in the IgG positive rate at 7-8 months, after which it was stable. The positive rate for vaccinated individuals was greater than that of unvaccinated individuals at 87.80% by 15-16 months.

The IgG antibody levels for all subjects showed a fluctuating upward trend over time(**Figure 2B**), from 5.20 to 12.85. There was a small peak at 11-12 months, when the IgG level reached 12.24. Remarkably, from 15-16 months onwards, a succession of patients had been vaccinated, it was evident that the IgG antibody levels were significantly higher in the vaccinated than in the unvaccinated (‘17-18 months’: 21.08 vs 9.32; ‘15-16 months’: 13.81 vs 9.15) participants.

### Evaluation of the Effectiveness of Vaccination on Convalescent Patients

**Figure 3:**
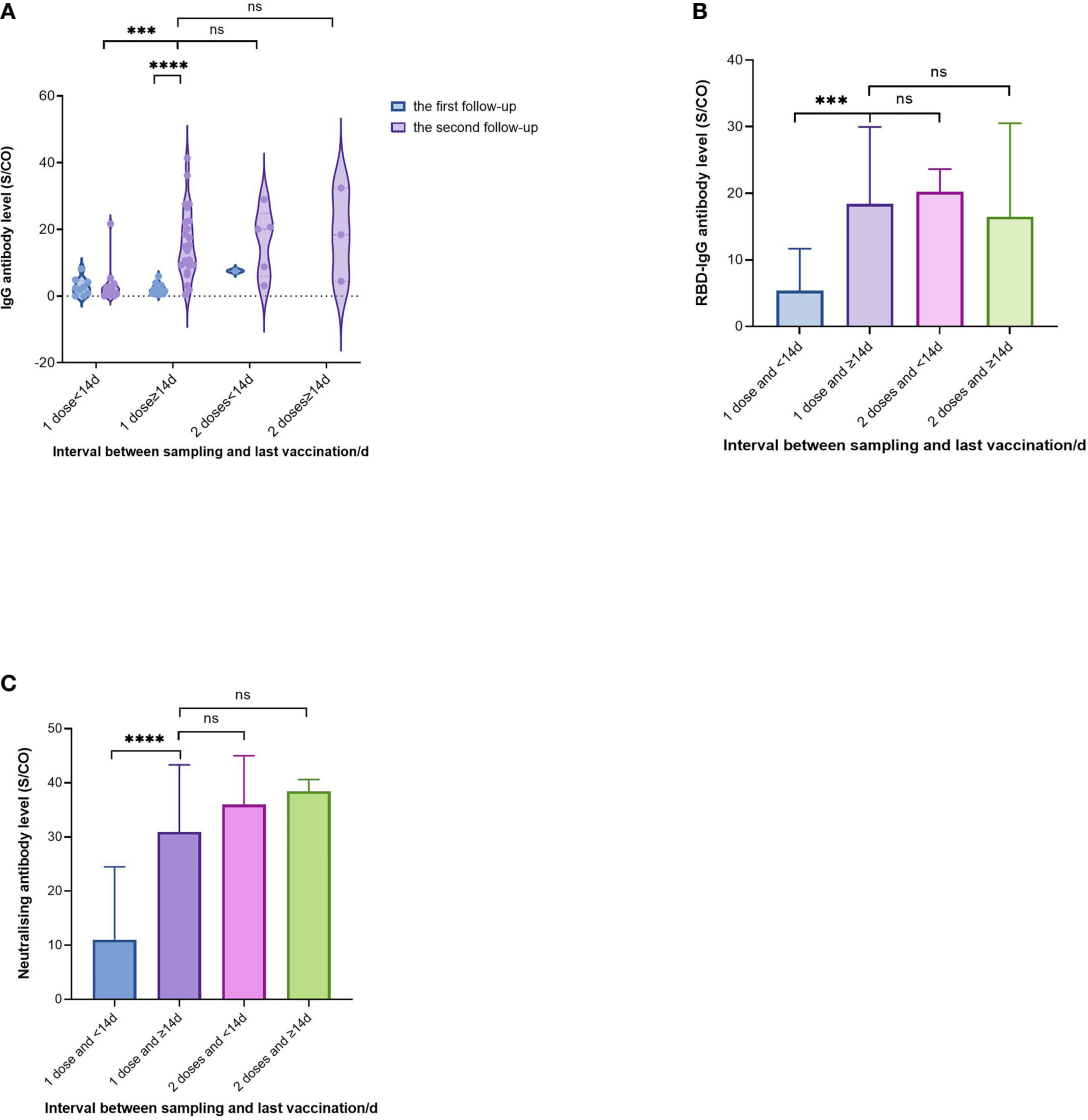
Evaluation of the effectiveness of vaccination on convalescent patients. The participants were divided into 4 parts according to the dose and the inoculation days between the sampling and the last vaccination: “1 dose and <14d”, “1 dose and ≥14d”, “2 doses and <14d” and “2 dose and ≥14d”. **(A)** Effect of different doses and inoculation days on IgG antibody levels in two rounds of follow-up. **(B)** Effect of different doses and inoculation days on RBD-IgG antibody levels. **(C)** Effect of different doses and inoculation days on neutralising antibody levels. P values were determined applying a two-tailed Mann-Whitney U test. P < 0.05 was considered to be statistically significant (ns: no significance; ***p < 0.001; ****p < 0.0001).

A total of 44 patients received an inactivated COVID-19 vaccine by the second round of follow-up. It was observed that the IgG levels were significantly higher after vaccination than those before vaccination in the same population (**Figure 3A**) by comparing the IgG levels in the first and second follow-up. The participants were divided into four parts according to the dose and the inoculation days between the sampling and the last vaccination: ‘1 dose and inoculation time less than 14 d’, ‘1 dose and ≥14 d’, ‘2 doses and <14 d’ and ‘2 dose and ≥14 d’ (**Figure 3**). IgG, RBD-IgG, and neutralizing antibodies were selected for evaluation. The comparison revealed that IgG levels in those who received ‘single dose and ≥14 d’ were significantly higher than those who received ‘single dose and <14 d’ (15.93 vs 3.55), and similar with those with ‘2 doses and <14 d’ (16.32) and ‘2 dose and ≥14 d’ (18.43) (*P*>0.05). Similar conclusions were drawn for RBD-IgG (**Figure 3B**) and neutralizing antibodies (**Figure 3C**).

## Discussion

The recent emergence of multiple viral variants and cases of re-infection have led to a reflection on the duration of the antibodies produced by the interaction of SARS-CoV-2 with the immune system. One of the key issues in the current COVID-19 pandemic is to understand the magnitude and kinetics of protective humoral immunity to SARS-CoV-2 following natural infection, which will undoubtedly contribute to protection against re-infection, public health policy development and vaccine progression (5, 11).

In this study, we conducted the second round of follow-up with 318 convalescent patients in Jiangsu Province (the longest follow-up was about 18 months). To the best of our knowledge, our cohort of patients with COVID-19 has the longest follow-up period worldwide (5). The first round of follow-up obtained the short-term kinetics of SARS-CoV-2 specific and neutralizing antibodies over 7 months after symptom onset in COVID-19 patients (3). The long-term characteristics of anti-SARS-CoV-2 antibodies were explored by examining the magnitude and trend of specific antibodies in these subjects over time. Vaccination information was also collected from these participants to understand the effect of vaccination on antibody levels. Our research found that IgG positive rates remained high (80.19%) and that vaccinated individuals had higher levels of IgG antibody compared to unvaccinated individuals. A similar conclusion can be drawn for IgG subtypes and neutralizing antibodies. These findings fill a gap in the kinetics of the long-term immune response to SARS-CoV-2 and highlight the need for vaccination of the convalescent population.

Previous studies have shown that antibodies caused by SARS-CoV-2 appear 3 days after the onset of symptoms or 1 week after infection (12, 13). Understanding population-level seroprevalence and humoral immune kinetics is essential for vaccination strategies (14). However, little is known about the durability of the long-term humoral response against SARS-CoV-2 (15). Dan et al. showed that the SARS-CoV-2 specific IgG antibody could be maintained for up to 8 months (16). He et al. found that participants maintained their anti-SARS-CoV-2 IgG antibodies for at least 9 months (15). Xiang et al. also found that most patients recovering from COVID-19 developed detectable SARS-CoV-2 specific IgG antibodies 1 year after the onset of symptoms (5). In our study, the rate of IgG positivity remained high at 80.19% during the almost 18 months of follow-up after symptom onset, and the IgG antibody level was 9.73 (95%CI, 8.28-11.19). Researchers have reported that IgG persisted for more than 2 years in patients recovering from SARS (17, 18). Since SARS-CoV-2 shares 79.6% genomic sequence homology with SARS-Cov (19), we assume that IgG has the potential to continue to exist. In addition, several studies have shown that anti-SARS-CoV-2 specific IgM levels decrease progressively over 3-5 months after infection (20). Our study found that 20.44% of participants were positive for IgM 13-14 months after the onset of symptoms, which complements the results of existing studies. A previous study found that RBD-IgG persisted in 96.8% (31 of 32) of subjects at 14 months (21), which was slightly higher than 89.31% (284/318) at 15.6 months after symptom onset in our study. We also found that vaccinated individuals had higher antibody levels of IgG, RBD-IgG, S-IgG, N-IgG and neutralizing antibodies than unvaccinated individuals, consistent with the conclusions reached by Carlos et al. (22).

This study also examined the relationship between positive rates and levels of specific antibodies, sex, age, and disease severity. Some reports have observed a correlation between antibody levels and male sex (23–25), which is consistent with our findings on IgG antibody levels in vaccinated individuals. However, we also found that the IgG-positive rate was slightly higher in females, which could be an illusion due to the small number of vaccinees and the uneven ratio of males to females, to be further verified by expanding the sample size. There was evidence that IgG antibody levels were positively correlated with age (11, 23, 24, 26), but we did not observe a significant difference in antibody levels between the age groups of 20-60 years and 60 years and above. In contrast, children and adolescents are more likely to be free of novel coronavirus or less symptomatic, and their immune response was not as violent as that of adults (27–30), consistent with our finding of lower antibody levels and positive rates in the <20 years age group. Compared to the other three clinical types, patients with the severe/critical type disease had higher antibody levels and positive rates, while those with the asymptomatic type disease had the lowest IgG levels and positive rates, which may be related to high levels of viral load or inflammatory storm in severe/critical patients (31). Our study further confirmed the findings of a previous study (32–35). We performed multivariate regression analyses of antibody positive rate and level with the factors, incorporating the cross effect between these factors such as sex, age, disease severity, and vaccination into the model. The interaction between the factors was ultimately confirmed to be non-existent.

The best indicator of vaccine protection is the epidemiological effectiveness of prevention. However, antibody levels can indirectly reflect the effectiveness of immune protection, as supported by Khoury et al. (36). A key strength of this study was a 15.6 months-long follow-up of a population recovering from natural infection with COVID-19, incorporating inactivated vaccination as a factor for the first time, and assessing changes in the seroprevalence and kinetics of anti-SARS-CoV-2 antibodies, including IgG, RBD-IgG, and neutralizing antibodies. Compared to unvaccinated individuals, IgG antibody levels of vaccinated individuals were elevated in overall, male, 30-40 age group, and asymptomatic and mild type groups; levels of RBD-IgG and neutralizing antibodies were increased in vaccinated individuals. These data illustrate the importance of improving vaccination uptake and aid in future COVID-19 public health measures. In addition, our study aimed to examine the effects of vaccination doses on vaccinated individuals. We found that in those with a single dose of the vaccine, IgG and neutralizing antibody levels were similar to those who received two doses, which serves as an effective immune booster (37, 38), supporting the notion that one dose is sufficient for patients with a novel coronavirus issued by the Technical Guidelines for Vaccination against Novel Coronavirus (1st Version) (39).

Our study had several limitations. First, a larger sample size is needed to draw more convincing conclusions. However, due to the need for continuous, prolonged follow-up of convalescent patients in this study, there was a lack of sufficient participants and loss to follow-up, resulting in an under-representation of severe/critical illness (n=4) and poor extrapolation of measurements. Presently, there is an increasing number of viral variants, and the extent and persistence of the human immune response to them has not yet been studied, a gap that needs to be addressed in future studies.

In summary, we found that most convalescent COVID-19 patients were still positive for IgG antibodies 15.6 months after symptom onset, which suggested the possibility of long-term immunization. People vaccinated with one dose of inactivated vaccine produced higher levels of antibodies than unvaccinated individuals, which was similar to those who received two doses. These findings can help governments and health authorities to implement more suitable vaccination strategies for people recovering from natural infections.

## Data Availability Statement

The raw data supporting the conclusions of this article will be made available by the authors, without undue reservation.

## Ethics Statement

The studies involving human participants were reviewed and approved by the Institutional Review Board of Jiangsu Provincial Center for Disease Control and Prevention (JSJK2021-B007-01). Written informed consent to participate in this study was provided by the participants’ legal guardian/next of kin.

## Author Contributions

SZ: Formal analysis, data curation, writing-original draft preparation, and writing-review and editing. KX, Investigation. CL, Laboratory detection. LZ, Laboratory detection. XK, Laboratory detection. JP, Laboratory detection. FZ, Supervision. CB, Supervision and funding. QG, Investigation. XZ, Investigation. HJ, Conceptualization and supervision. LGZ: Investigation, Resources, Project Administration, Writing-Original Draft Preparation, and Writing-Review and Editing. All authors contributed to the article and approved the submitted version.

## Funding

This work was supported by Postgraduate Research and Practice Innovation Program of Jiangsu Province under grant KYCX20_0153; Jiangsu Provincial Six Talent Peak under grant number: WSN-002; National Natural Science Foundation of China under grant number 82041026; Social Development Foundation of Jiangsu Province under grant number BE2021739.

## Conflict of Interest

The authors declare that the research was conducted in the absence of any commercial or financial relationships that could be construed as a potential conflict of interest.

## Publisher’s Note

All claims expressed in this article are solely those of the authors and do not necessarily represent those of their affiliated organizations, or those of the publisher, the editors and the reviewers. Any product that may be evaluated in this article, or claim that may be made by its manufacturer, is not guaranteed or endorsed by the publisher.
