# Supplementary material for: Long-Term Kinetics of SARS-CoV-2 Antibodies and Impact of Inactivated Vaccine on SARS-CoV-2 Antibodies Based on a COVID-19 Patients Cohort
Source: Front Immunol. 2022 Jan 27;13:829665. doi: 10.3389/fimmu.2022.829665 (PMC8828498; doi:10.3389/fimmu.2022.829665)
Supplement: Supplementary file 1 [file DataSheet_1.docx]

| **Table S1**  Distribution of IgM/IgG positive rate and antibody levels among non-vaccinated convalescent patients. | | | | | | | | | |
| --- | --- | --- | --- | --- | --- | --- | --- | --- | --- |
|  | Subjects | IgG positive number  (%) | *P* | IgG antibody levels (mean,95%CI) | *P* | IgM positive number  (%) | *P* | IgM  antibody levels  (mean,95%CI) | *P* |
|  | 274 | 216(78.83) |  | 8.90(7.32,10.48) |  | 53(19.34) |  | 4.98(2.54,7.41) |  |
| Sex |  |  | 0.585 |  | 0.424 |  | 0.824 |  | 0.678 |
| Male | 133 | 103(77.44) |  | 8.22(5.93,10.52) |  | 25(18.80) |  | 4.44(1.72,7.16) |  |
| Female | 141 | 113(80.14) |  | 9.51(7.33,11.69) |  | 28(19.86) |  | 5.46(1.50,9.41) |  |
| Age (ys) |  |  | 0.017^*^ |  | 0.415 |  | 0.735 |  | 0.102 |
| <20 | 17 | 9(52.94) |  | 4.70(2.57,6.82) |  | 1(5.88) |  | 1.44 |  |
| 20~ | 28 | 19(67.86) |  | 10.98(2.41,19.55) |  | 6(21.43) |  | 2.07(0.78,3.37) |  |
| 30~ | 64 | 49(76.56) |  | 6.49(4.80,8.18) |  | 14(21.88) |  | 2.47(1.63,3.31) |  |
| 40~ | 44 | 37(84.09) |  | 9.73(6.81,12.66) |  | 10(22.73) |  | 4.48(1.81,7.14) |  |
| 50~ | 64 | 57(89.06) |  | 10.51(6.31,14.71) |  | 12(18.75) |  | 11.53(1.81,21.25) |  |
| ≥60 | 57 | 45(78.95) |  | 8.76(6.15,11.36) |  | 10(17.54) |  | 3.22(1.67,4.78) |  |
| Severity of disease |  |  | 0.036^*^ |  | 0.291 |  | 0.186 |  | 0.522 |
| Asymtomatic type | 82 | 56(68.29) |  | 6.62(4.90,8.33) |  | 16(19.51) |  | 3.30(1.62,4.99) |  |
| Mild type | 46 | 37(80.43) |  | 8.28(5.61,10.96) |  | 7(15.22) |  | 2.90(1.05,4.75) |  |
| Normal type | 143 | 120(83.92) |  | 10.20(7.62,12.79) |  | 28(19.58) |  | 6.69(2.27,11.11) |  |
| Severe/Critical type | 3 | 3(100.00) |  | 6.93(2.60,11.26) |  | 2(66.67) |  | 1.61(0.92,2.31) |  |
| P < 0.05 represents significant difference. *p<0.05; **p<0.01; ***p<0.001; ****p<0.0001 | | | | | | | | | |

| **Table S2** Distribution of IgM/IgG positive rate and antibody levels among vaccinated convalescent patients. | | | | | | | | | |
| --- | --- | --- | --- | --- | --- | --- | --- | --- | --- |
|  | Subjects | IgG positive number  (%) | *P* | IgG antibody levels (mean,95%CI) | *P* | IgM positive number  (%) | *P* | IgM  antibody levels  (mean,95%CI) | *P* |
|  | 44 | 39(88.64) |  | 14.37(10.92,17.81) |  | 12(27.27) |  | 4.31(1.95,6.68) |  |
| Sex |  |  | 0.044^*^ |  | 0.270 |  | 0.658 |  | 0.318 |
| Male | 17 | 13(76.47) |  | 17.05(10.40,23.70) |  | 4(23.53) |  | 2.72(0.92,4.53) |  |
| Female | 27 | 26(96.30) |  | 13.03(9.08,16.97) |  | 8(29.63) |  | 5.11(1.76,8.46) |  |
| Age (ys) |  |  | 0.369 |  | 0.464 |  | 0.224 |  | 0.332 |
| <20 | 0 | 0 |  | - |  | 0 |  | - |  |
| 20~ | 4 | 4(100.00) |  | 8.83(0.12,17.54) |  | 2(50.00) |  | 4.01(-0.65,8.67) |  |
| 30~ | 15 | 12(80.00) |  | 11.82(7.09,16.56) |  | 3(20.00) |  | 8.17(0.79,15.54) |  |
| 40~ | 10 | 8(80.00) |  | 15.24(6.07,24.42) |  | 5(50.00) |  | 2.27(0.79,3.75) |  |
| 50~ | 10 | 10(100.00) |  | 18.98(11.39,26.56) |  | 1(10.00) |  | 4.84 |  |
| ≥60 | 5 | 5(100.00) |  | 14.29(4.04,24.54) |  | 1(20.00) |  | 3.07 |  |
| Severity of disease |  |  | 0.012^*^ |  | 0.839 |  | 0.308 |  | 0.006^**^ |
| Asymtomatic type | 10 | 6(60.00) |  | 14.02(2.16,25.89) |  | 1(10.00) |  | 13.44 |  |
| Mild type | 14 | 13(92.86) |  | 15.88(10.60,21.16) |  | 6(42.86) |  | 4.50(1.77,7.24) |  |
| Normal type | 19 | 19(100.00) |  | 13.11(8.09,18.12) |  | 5(26.32) |  | 2.27(1.54,2.99) |  |
| Severe/Critical type | 1 | 1(100.00) |  | 20.71 |  | 0 |  | - |  |
| P < 0.05 represents significant difference. *p<0.05; **p<0.01; ***p<0.001; ****p<0.0001 | | | | | | | | | |

| **Table S3** Comparisons of IgG positive rates between those convalescent patients with and without vaccination. | | | |
| --- | --- | --- | --- |
|  | Non-vaccinated | Vaccinated | *P* |
| Overall | 216/274(78.83) | 39/44(88.64) | 0.130 |
| Sex |  |  |  |
| Male | 103/133(77.44) | 13/17(76.47) | 0.928 |
| Female | 113/141(80.14) | 26/27(96.30) | 0.042^*^ |
| Age (ys) |  |  |  |
| <20 | 9/17(52.94) | 0 | - |
| 20~ | 19/28(67.86) | 4/4(100.00) | 0.303 |
| 30~ | 49/64(76.56) | 12/15(80.00) | 0.775 |
| 40~ | 37/44(84.09) | 8/10(80.00) | 0.754 |
| 50~ | 57/64(89.06) | 10/10(100.00) | 0.583 |
| ≥60 | 45/57(78.95) | 5/5(100.00) | 0.573 |
| Severity of disease |  |  |  |
| Asymtomatic type | 56/82(68.29) | 6/10(60.00) | 0.597 |
| Mild type | 37/46(80.43) | 13/14(92.86) | 0.275 |
| Normal type | 120/143(83.92) | 19/19(100.00) | 0.059 |
| Severe/Critical type | 3/3(100.00) | 1/1(100.00) | - |
| P < 0.05 represents significant difference. *p<0.05; **p<0.01; ***p<0.001; ****p<0.0001 | | | |

| **Table S4** Comparisons of IgG antibody levels between those convalescent patients with and without vaccination. | | | |
| --- | --- | --- | --- |
|  | Non-vaccinated | Vaccinated | *P* |
| Overall | 8.90(7.32,10.48) | 14.37(10.92,17.81) | 0.007^**^ |
| Sex |  |  |  |
| Male | 8.22(5.93,10.52) | 17.05(10.40,23.70) | 0.013^*^ |
| Female | 9.51(7.33,11.69) | 13.03(9.08,16.97) | 0.161 |
| Age (ys) |  |  |  |
| <20 | 4.70(2.57,6.82) | - | - |
| 20~ | 10.98(2.41,19.55) | 8.83(0.12,17.54) | 0.829 |
| 30~ | 6.49(4.80,8.18) | 11.82(7.09,16.56) | 0.013^*^ |
| 40~ | 9.73(6.81,12.66) | 15.24(6.07,24.42) | 0.155 |
| 50~ | 10.51(6.31,14.71) | 18.98(11.39,26.56) | 0.118 |
| ≥60 | 8.76(6.15,11.36) | 14.29(4.04,24.54) | 0.204 |
| Severity of disease |  |  |  |
| Asymtomatic type | 6.62(4.90,8.33) | 14.02(2.16,25.89) | 0.025^*^ |
| Mild type | 8.28(5.61,10.96) | 15.88(10.60,21.16) | 0.008^**^ |
| Normal type | 10.20(7.62,12.79) | 13.11(8.09,18.12) | 0.400 |
| Severe/Critical type | 6.93(2.60,11.26) | 20.71 | - |
| P < 0.05 represents significant difference. *p<0.05; **p<0.01; ***p<0.001; ****p<0.0001 | | | |

| **Table S5** Comparisons of IgM positive rates between those convalescent patients with and without vaccination. | | | |
| --- | --- | --- | --- |
|  | Non-vaccinated | Vaccinated | *P* |
| Overall | 53/274(19.34) | 12/44(27.27) | 0.226 |
| Sex |  |  |  |
| Male | 25/133(18.80) | 4/17(23.53) | 0.642 |
| Female | 28/141(19.86) | 8/27(29.63) | 0.257 |
| Age (ys) |  |  |  |
| <20 | 1/17(5.88) | 0 | - |
| 20~ | 6/28(21.43) | 2/4(50.00) | 0.254 |
| 30~ | 14/64(21.88) | 3/15(20.00) | 0.874 |
| 40~ | 10/44(22.73) | 5/10(50.00) | 0.082 |
| 50~ | 12/64(18.75) | 1/10(10.00) | 0.499 |
| ≥60 | 10/57(17.54) | 1/5(20.00) | 1.000 |
| Severity of disease |  |  |  |
| Asymtomatic type | 16/82(19.51) | 1/10(10.00) | 0.464 |
| Mild type | 7/46(15.22) | 6/14(42.86) | 0.028^*^ |
| Normal type | 28/143(19.58) | 5/19(26.32) | 0.493 |
| Severe/Critical type | 2/3(66.67) | 0 | 1.000 |
| P < 0.05 represents significant difference. *p<0.05; **p<0.01; ***p<0.001; ****p<0.0001 | | | |

| **Table S6** Comparisons of IgM antibody levels between those convalescent patients with and without vaccination. | | | |
| --- | --- | --- | --- |
|  | Non-vaccinated | Vaccinated | *P* |
| Overall | 4.98(2.54,7.41) | 4.31(1.95,6.68) | 0.801 |
| Sex |  |  |  |
| Male | 4.44(1.72,7.16) | 2.72(0.92,4.53) | 0.624 |
| Female | 5.46(1.50,9.41) | 5.11(1.76,8.46) | 0.927 |
| Age (ys) |  |  |  |
| <20 | 1.44 | - | - |
| 20~ | 2.07(0.78,3.37) | 4.01(-0.65,8.67) | 0.256 |
| 30~ | 2.47(1.63,3.31) | 8.17(0.79,15.54) | 0.003^**^ |
| 40~ | 4.48(1.81,7.14) | 2.27(0.79,3.75) | 0.282 |
| 50~ | 11.53(1.81,21.25) | 4.84 | - |
| ≥60 | 3.22(1.67,4.78) | 3.07 | - |
| Severity of disease |  |  |  |
| Asymtomatic type | 3.30(1.62,4.99) | 13.44 | - |
| Mild type | 2.90(1.05,4.75) | 4.50(1.77,7.24) | 0.313 |
| Normal type | 6.69(2.27,11.11) | 2.27(1.54,2.99) | 0.408 |
| Severe/Critical type | 1.61(0.92,2.31) | - | - |
| P < 0.05 represents significant difference. *p<0.05; **p<0.01; ***p<0.001; ****p<0.0001 | | | |

| **Table S7 Distribution of IgG subtypes and neutralizing antibodies positive rate and antibody levels among the non-vaccinated convalescent patients.** | | | | | | | | | | | | | | | | |
| --- | --- | --- | --- | --- | --- | --- | --- | --- | --- | --- | --- | --- | --- | --- | --- | --- |
|  | **RBD-IgG positive number(%)** | **P** | **S-IgG positive number(%)** | **P** | **N-IgG positive number(%)** | **P** | **Nab positive number(%)** | **P** | **RBD-IgG antibody levels (mean,95%CI)** | **P** | **S-IgG antibody levels (mean,95%CI)** | **P** | **N-IgG antibody levels (mean,95%CI)** | **P** | **Nab**  **antibody levels (mean,95%CI)** | **P** |
| Unvaccinated | 244(89.05) |  | 251(91.61) |  | 213(77.74) |  | 225(82.12) |  | 10.54(9.06,12.02) |  | 13.86(11.99,15.74) |  | 7.33(6.03,8.62) |  | 16.11(14.21,18.01) |  |
| Sex |  | 0.345 |  | 0.424 |  | 0.686 |  | 0.946 |  | 0.494 |  | 0.338 |  | 0.867 |  | 0.294 |
| Male | 116(87.22) |  | 120(90.23) |  | 102(76.69) |  | 109(81.95) |  | 10.00(7.77,12.24) |  | 12.91(10.17,15.65) |  | 7.21(5.53,8.89) |  | 15.06(12.44,17.69) |  |
| Female | 128(90.78) |  | 131(92.91) |  | 111(78.72) |  | 116(82.27) |  | 11.03(9.04,13.02) |  | 14.74(12.15,17.33) |  | 7.43(5.45,9.41) |  | 17.09(14.33,19.86) |  |
| Age (ys) |  | 0.778 |  | 0.626 |  | 0.001^**^ |  | 0.682 |  | 0.271 |  | 0.147 |  | 0.004^**^ |  | 0.943 |
| <20 | 15(88.24) |  | 15(88.24) |  | 7(41.18) |  | 14(82.35) |  | 5.85(2.57,9.12) |  | 8.26(3.56,12.96) |  | 3.39(1.70,5.07) |  | 12.67(4.61,20.72) |  |
| 20~ | 24(85.71) |  | 24(85.71) |  | 18(64.29) |  | 22(78.57) |  | 11.66(4.49,18.84) |  | 14.55(6.26,22.83) |  | 4.02(2.25,5.79) |  | 15.46(8.36,22.55) |  |
| 30~ | 55(85.94) |  | 57(89.06) |  | 48(75.00) |  | 50(78.13) |  | 8.82(6.47,11.16) |  | 10.74(8.01,13.47) |  | 4.28(3.55,5.00) |  | 16.28(12.11,20.46) |  |
| 40~ | 41(93.18) |  | 41(93.18) |  | 38(86.36) |  | 36(81.82) |  | 9.75(7.31,12.20) |  | 13.22(10.17,16.27) |  | 8.65(5.28,12.02) |  | 17.56(13.05,22.08) |  |
| 50~ | 59(92.19) |  | 61(95.31) |  | 54(84.38) |  | 57(89.06) |  | 12.81(8.76,16.85) |  | 17.31(12.20,22.43) |  | 11.03(7.02,15.03) |  | 16.33(12.40,20.26) |  |
| ≥60 | 50(87.72) |  | 53(92.98) |  | 48(84.21) |  | 46(80.70) |  | 11.29(8.40,14.17) |  | 15.02(10.94,19.11) |  | 6.97(5.01,8.93) |  | 15.86(11.52,20.21) |  |
| Severity of disease |  | 0.023^*^ |  | 0.069 |  | 0.001^**^ |  | 0.044^*^ |  | 0.308 |  | 0.288 |  | 0.966 |  | 0.183 |
| Asymtomatic type | 66(80.49) |  | 70(85.37) |  | 51(62.20) |  | 60(73.17) |  | 8.33(6.23,10.44) |  | 10.93(8.10,13.76) |  | 6.84(4.43,9.24) |  | 12.96(9.79,16.12) |  |
| Mild type | 44(95.65) |  | 45(97.83) |  | 40(86.96) |  | 42(91.30) |  | 10.42(7.77,13.06) |  | 14.46(10.85,18.07) |  | 7.64(5.41,9.86) |  | 15.55(10.94,20.15) |  |
| Normal type | 131(91.61) |  | 133(93.01) |  | 119(83.22) |  | 120(83.92) |  | 11.69(9.30,14.09) |  | 15.14(12.17,18.12) |  | 7.39(5.42,9.36) |  | 17.75(15.00,20.49) |  |
| Severe/Critical type | 3(100.00) |  | 3(100.00) |  | 3(100.00) |  | 3(100.00) |  | 10.91(3.11,18.72) |  | 16.61(-7.96,41.17) |  | 8.98(-8.47,26.42) |  | 21.37(-21.34,64.08) |  |
| Months after symptom onset |  | 0.038^*^ |  | 0.148 |  | 0.027^*^ |  | 0.004^**^ |  | 0.225 |  | 0.284 |  | 0.843 |  | 0.037^*^ |
| 9~10M | 8(100.00) |  | 8(100.00) |  | 8(100.00) |  | 8(100.00) |  | 5.43(1.46,9.40) |  | 8.55(2.56,14.54) |  | 5.82(1.33,10.32) |  | 13.83(1.97,25.69) |  |
| 11~12M | 7(63.64) |  | 8(72.73) |  | 5(45.45) |  | 6(54.55) |  | 17.91(0.28,35.54) |  | 21.82(0.02,43.62) |  | 10.46(0.37,20.55) |  | 13.69(-0.95,28.34) |  |
| 13~14M | 64(86.49) |  | 67(90.54) |  | 57(77.03) |  | 54(72.97) |  | 9.05(6.92,11.18) |  | 11.89(9.20,14.58) |  | 8.18(5.43,10.93) |  | 12.82(9.28,16.36) |  |
| 15~16M | 158(90.80) |  | 161(92.53) |  | 136(78.16) |  | 150(86.21) |  | 11.02(9.02,13.02) |  | 14.38(11.87,16.89) |  | 6.99(5.34,8.64) |  | 16.86(14.48,19.23) |  |
| 17~18M | 7(100.00) |  | 7(100.00) |  | 7(100.00) |  | 7(100.00) |  | 12.02(5.11,18.94) |  | 17.78(7.07,28.49) |  | 6.32(2.34,10.30) |  | 30.13(16.57,43.69) |  |
| *P* < 0.05 represents significant difference. *p<0.05; **p<0.01; ***p<0.001; ****p<0.0001 | | | | | | | | | | | | | | | | |

| **Table S8 Distribution of IgG subtypes and neutralizing antibodies positive rate and antibody levels among the vaccinated convalescent patients.** | | | | | | | | | | | | | | | | |
| --- | --- | --- | --- | --- | --- | --- | --- | --- | --- | --- | --- | --- | --- | --- | --- | --- |
|  | **RBD-IgG positive number(%)** | **P** | **S-IgG positive number(%)** | **P** | **N-IgG positive number(%)** | **P** | **Nab**  **positive number(%)** | **P** | **RBD-IgG antibody levels (mean,95%CI)** | **P** | **S-IgG antibody levels (mean,95%CI)** | **P** | **N-IgG antibody levels (mean,95%CI)** | **P** | **Nab**  **antibody levels (mean,95%CI)** | **P** |
| Vaccinated | 40(90.91) |  | 40(90.91) |  | 38(86.36) |  | 39(88.64) |  | 16.41(12.97,19.85) |  | 24.11(18.81,29.40) |  | 18.50(12.60,24.40) |  | 29.83(25.62,34.04) |  |
| Sex |  | 0.117 |  | 0.117 |  | 0.774 |  | 0.044 |  | 0.152 |  | 0.129 |  | 0.326 |  | 0.281 |
| Male | 14(82.35) |  | 14(82.35) |  | 15(88.24) |  | 13(76.47) |  | 19.75(11.59,27.92) |  | 29.55(16.53,42.56) |  | 22.09(8.56,35.61) |  | 33.04(26.66,39.43) |  |
| Female | 26(96.30) |  | 26(96.30) |  | 23(85.19) |  | 26(96.30) |  | 14.61(11.30,17.92) |  | 21.18(16.43,25.92) |  | 16.16(10.91,21.40) |  | 28.22(22.58,33.86) |  |
| Age (ys) |  | 0.397 |  | 0.397 |  | 0.433 |  | 0.369 |  | 0.325 |  | 0.517 |  | 0.065 |  | 0.437 |
| <20 | 0 |  | 0 |  | 0 |  | 0 |  | - |  | - |  | - |  | - |  |
| 20~ | 4(100.00) |  | 4(100.00) |  | 4(100.00) |  | 4(100.00) |  | 10.37(0.42,20.33) |  | 13.64(0.93,26.35) |  | 12.52(-3.25,28.28) |  | 21.16(-3.63,45.95) |  |
| 30~ | 12(80.00) |  | 12(80.00) |  | 11(73.33) |  | 12(80.00) |  | 14.47(8.14,20.80) |  | 21.31(12.65,29.98) |  | 11.37(6.04,16.71) |  | 29.45(20.88,38.02) |  |
| 40~ | 9(90.00) |  | 9(90.00) |  | 9(90.00) |  | 8(80.00) |  | 16.93(5.38,28.49) |  | 25.45(5.22,45.67) |  | 13.27(6.61,19.93) |  | 26.88(14.35,39.41) |  |
| 50~ | 10(100.00) |  | 10(100.00) |  | 9(90.00) |  | 10(100.00) |  | 22.04(15.23,28.86) |  | 30.23(21.06,39.40) |  | 26.42(9.07,43.77) |  | 34.88(27.79,41.96) |  |
| ≥60 | 5(100.00) |  | 5(100.00) |  | 5(100.00) |  | 5(100.00) |  | 13.69(6.42,20.96) |  | 24.51(10.38,38.64) |  | 34.10(-3.06,71.27) |  | 32.29(17.40,47.18) |  |
| Severity of disease |  | 0.061 |  | 0.061 |  | 0.038^*^ |  | 0.012^*^ |  | 0.711 |  | 0.796 |  | 0.752 |  | 0.818 |
| Asymtomatic type | 7(70.00) |  | 7(70.00) |  | 6(60.00) |  | 6(60.00) |  | 12.80(1.86,23.74) |  | 18.78(3.94,33.62) |  | 12.71(1.72,23.70) |  | 28.98(13.16,44.79) |  |
| Mild type | 13(92.86) |  | 13(92.86) |  | 14(100.00) |  | 13(92.86) |  | 18.80(13.01,24.58) |  | 26.47(19.16,33.78) |  | 20.39(7.29,33.49) |  | 31.71(23.43,39.99) |  |
| Normal type | 19(100.00) |  | 19(100.00) |  | 17(89.47) |  | 19(100.00) |  | 16.16(10.58,21.74) |  | 24.19(14.62,33.77) |  | 18.25(9.85,26.64) |  | 28.35(22.28,34.43) |  |
| Severe/Critical type | 1(100.00) |  | 1(100.00) |  | 1(100.00) |  | 1(100.00) |  | 15.41 |  | 28.97 |  | 30.98 |  | 38.46 |  |
| Months after symptom onset |  | 0.570 |  | 0.570 |  | 0.476 |  | 0.521 |  | 0.878 |  | 0.360 |  | 0.653 |  | 0.145 |
| 15~16M | 37(90.24) |  | 37(90.24) |  | 35(85.37) |  | 36(87.80) |  | 16.33(12.62,20.05) |  | 23.41(17.78,29.05) |  | 18.11(11.75,24.46) |  | 28.95(24.50,33.39) |  |
| 17~18M | 3(100.00) |  | 3(100.00) |  | 3(100.00) |  | 3(100.00) |  | 17.35(5.20,29.50) |  | 32.65(9.80,55.50) |  | 23.05(-2.66,48.76) |  | 40.4(36.00,44.80) |  |
| *P* < 0.05 represents significant difference. *p<0.05; **p<0.01; ***p<0.001; ****p<0.0001 | | | | | | | | | | | | | | | | |

| **Table S9 Comparisons of RBD-IgG positive rate and antibody levels between those convalescent patients with and without vaccination.** | | | | | | | |
| --- | --- | --- | --- | --- | --- | --- | --- |
| **RBD-IgG positive number (%)** | | | | **RBD-IgG antibody levels (mean,95%CI)** | | | |
|  | Unvaccinated | Vaccinated | P |  | Unvaccinated | Vaccinated | P |
| Overall | 244/274(89.05) | 40/44(90.91) | 0.711 | Overall | 10.54(9.06,12.02) | 16.41(12.97,19.85) | 0.003^**^ |
| Sex |  |  |  | Sex |  |  |  |
| Male | 116/133(87.22) | 14/17(82.35) | 0.578 | Male | 10.00(7.77,12.24) | 19.75(11.59,27.92) | 0.006^**^ |
| Female | 128/141(90.78) | 26/27(96.30) | 0.342 | Female | 11.03(9.04,13.02) | 14.61(11.30,17.92) | 0.130 |
| Age (ys) |  |  |  | Age (ys) |  |  |  |
| <20 | 15/17(88.24) | 0 | - | <20 | 5.85(2.57,9.12) | - | - |
| 20~ | 24/28(85.71) | 4/4(100.00) | 1.000 | 20~ | 11.66(4.49,18.84) | 10.37(0.42,20.33) | 0.883 |
| 30~ | 55/64(85.94) | 12/15(80.00) | 0.564 | 30~ | 8.82(6.47,11.16) | 14.47(8.14,20.80) | 0.051 |
| 40~ | 41/44(93.18) | 9/10(90.00) | 0.729 | 40~ | 9.75(7.31,12.20) | 16.93(5.38,28.49) | 0.043^*^ |
| 50~ | 59/64(92.19) | 10/10(100.00) | 0.360 | 50~ | 12.81(8.76,16.85) | 22.04(15.23,28.86) | 0.074 |
| ≥60 | 50/57(87.72) | 5/5(100.00) | 0.405 | ≥60 | 11.29(8.40,14.17) | 13.69(6.42,20.96) | 0.607 |
| Severity of disease |  |  |  | Severity of disease |  |  |  |
| Asymtomatic type | 66/82(80.49) | 7/10(70.00) | 0.439 | Asymtomatic type | 8.33(6.23,10.44) | 12.80(1.86,23.74) | 0.210 |
| Mild type | 44/46(95.65) | 13/14(92.86) | 0.674 | Mild type | 10.42(7.77,13.06) | 18.80(13.01,24.58) | 0.004^**^ |
| Normal type | 131/143(91.61) | 19/19(100.00) | 0.189 | Normal type | 11.69(9.30,14.09) | 16.16(10.58,21.74) | 0.183 |
| Severe/Critical type | 3/3(100.00) | 1/1(100.00) | - | Severe/Critical type | 10.91(3.11,18.72) | 15.41 | - |
| Months after symptom onset |  |  |  | Months after symptom onset |  |  |  |
| 15~16M | 158/174(90.80) | 37/41(90.24) | 0.911 | 15~16M | 11.02(9.02,13.02) | 16.33(12.62,20.05) | 0.020^*^ |
| 17~18M | 7/7(100.00) | 3/3(100.00) | - | 17~18M | 12.02(5.11,18.94) | 17.35(5.20,29.50) | 0.298 |
| *P* < 0.05 represents significant difference. *p<0.05; **p<0.01; ***p<0.001; ****p<0.0001 | | | | | | | |

| **Table S10 Comparisons of S-IgG positive rate and antibody levels between those convalescent patients with and without vaccination.** | | | | | | | |
| --- | --- | --- | --- | --- | --- | --- | --- |
| **S-IgG positive number (%)** | | | | **S-IgG antibody levels (mean,95%CI)** | | | |
|  | Unvaccinated | Vaccinated | P |  | Unvaccinated | Vaccinated | P |
| Overall | 251/274(91.61) | 40/44(90.91) | 0.878 | Overall | 13.86(11.99,15.74) | 24.11(18.81,29.40) | <0.001^***^ |
| Sex |  |  |  | Sex |  |  |  |
| Male | 120/133(90.23) | 14/17(82.35) | 0.322 | Male | 12.91(10.17,15.65) | 29.55(16.53,42.56) | <0.001^***^ |
| Female | 131/141(92.91) | 26/27(96.30) | 0.514 | Female | 14.74(12.15,17.33) | 21.18(16.43,25.92) | 0.041^*^ |
| Age (ys) |  |  |  | Age (ys) |  |  |  |
| <20 | 15/17(88.24) | 0 | - | <20 | 8.26(3.56,12.96) | - | - |
| 20~ | 24/28(85.71) | 4/4(100.00) | 1.000 | 20~ | 14.55(6.26,22.83) | 13.64(0.93,26.35) | 0.929 |
| 30~ | 57/64(89.06) | 12/15(80.00) | 0.342 | 30~ | 10.74(8.01,13.47) | 21.31(12.65,29.98) | 0.003^**^ |
| 40~ | 41/44(93.18) | 9/10(90.00) | 0.571 | 40~ | 13.22(10.17,16.27) | 25.45(5.22,45.67) | 0.021^*^ |
| 50~ | 61/64(95.31) | 10/10(100.00) | 1.000 | 50~ | 17.31(12.20,22.43) | 30.23(21.06,39.40) | 0.053 |
| ≥60 | 53/57(92.98) | 5/5(100.00) | 1.000 | ≥60 | 15.02(10.94,19.11) | 24.51(10.38,38.64) | 0.171 |
| Severity of disease |  |  |  | Severity of disease |  |  |  |
| Asymtomatic type | 70/82(85.37) | 7/10(70.00) | 0.214 | Asymtomatic type | 10.93(8.10,13.76) | 18.78(3.94,33.62) | 0.111 |
| Mild type | 45/46(97.83) | 13/14(92.86) | 0.364 | Mild type | 14.46(10.85,18.07) | 26.47(19.16,33.78) | 0.003^**^ |
| Normal type | 133/143(93.01) | 19/19(100.00) | 0.234 | Normal type | 15.14(12.17,18.12) | 24.19(14.62,33.77) | 0.038^*^ |
| Severe/Critical type | 3/3(100.00) | 1/1(100.00) | - | Severe/Critical type | 16.61(-7.96,41.17) | 28.97 | - |
| Months after symptom onset |  |  |  | Months after symptom onset |  |  |  |
| 15~16M | 161/174(92.53) | 37/41(90.24) | 0.626 | 15~16M | 14.38(11.87,16.89) | 23.41(17.78,29.05) | 0.003^**^ |
| 17~18M | 7/7(100.00) | 3/3(100.00) | - | 17~18M | 17.78(7.07,28.49) | 32.65(9.80,55.50) | 0.087 |
| *P* < 0.05 represents significant difference. *p<0.05; **p<0.01; ***p<0.001; ****p<0.0001 | | | | | | | |

| **Table S11 Comparisons of N-IgG positive rate and antibody levels between those convalescent patients with and without vaccination.** | | | | | | | |
| --- | --- | --- | --- | --- | --- | --- | --- |
| **N-IgG positive number (%)** | | | | **N-IgG antibody levels (mean,95%CI)** | | | |
|  | Unvaccinated | Vaccinated | P |  | Unvaccinated | Vaccinated | P |
| Overall | 213/274(77.74) | 38/44(86.36) | 0.193 | Overall | 7.33(6.03,8.62) | 18.50(12.60,24.40) | <0.001*** |
| Sex |  |  |  | Sex |  |  |  |
| Male | 102/133(76.69) | 15/17(88.24) | 0.279 | Male | 7.21(5.53,8.89) | 22.09(8.56,35.61) | <0.001^***^ |
| Female | 111/141(78.72) | 23/27(85.19) | 0.444 | Female | 7.43(5.45,9.41) | 16.16(10.91,21.40) | <0.001^***^ |
| Age (ys) |  |  |  | Age (ys) |  |  |  |
| <20 | 7/17(41.18) | 0 | - | <20 | 3.39(1.70,5.07) | - | - |
| 20~ | 18/28(64.29) | 4/4(100.00) | 0.283 | 20~ | 4.02(2.25,5.79) | 12.52(-3.25,28.28) | 0.006^**^ |
| 30~ | 48/64(75.00) | 11/15(73.33) | 0.894 | 30~ | 4.28(3.55,5.00) | 11.37(6.04,16.71) | <0.001^***^ |
| 40~ | 38/44(86.36) | 9/10(90.00) | 0.757 | 40~ | 8.65(5.28,12.02) | 13.27(6.61,19.93) | 0.219 |
| 50~ | 54/64(84.38) | 9/10(90.00) | 0.642 | 50~ | 11.03(7.02,15.03) | 26.42(9.07,43.77) | 0.009^**^ |
| ≥60 | 48/57(84.21) | 5/5(100.00) | 1.000 | ≥60 | 6.97(5.01,8.93) | 34.10(-3.06,71.27) | <0.001^***^ |
| Severity of disease |  |  |  | Severity of disease |  |  |  |
| Asymtomatic type | 51/82(62.20) | 6/10(60.00) | 0.893 | Asymtomatic type | 6.84(4.43,9.24) | 12.71(1.72,23.70) | 0.125 |
| Mild type | 40/46(86.96) | 14/14(100.00) | 0.154 | Mild type | 7.64(5.41,9.86) | 20.39(7.29,33.49) | 0.002^**^ |
| Normal type | 119/143(83.22) | 17/19(89.47) | 0.485 | Normal type | 7.39(5.42,9.36) | 18.25(9.85,26.64) | <0.001^***^ |
| Severe/Critical type | 3/3(100.00) | 1/1(100.00) | - | Severe/Critical type | 8.98(-8.47,26.42) | 30.98 | - |
| Months after symptom onset |  |  |  | Months after symptom onset |  |  |  |
| 15~16M | 136/174(78.16) | 35/41(85.37) | 0.304 | 15~16M | 6.99(5.34,8.64) | 18.11(11.75,24.46) | <0.001^***^ |
| 17~18M | 7/7(100.00) | 3/3(100.00) | - | 17~18M | 6.32(2.34,10.30) | 23.05(-2.66,48.76) | 0.005^**^ |
| *P* < 0.05 represents significant difference. *p<0.05; **p<0.01; ***p<0.001; ****p<0.0001 | | | | | | | |

| **Table S12 Comparisons of Nab positive rate and antibody levels between those convalescent patients with and without vaccination.** | | | | | | | |
| --- | --- | --- | --- | --- | --- | --- | --- |
| **Nab positive number (%)** | | | | **Nab antibody levels (mean,95%CI)** | | | |
|  | Unvaccinated | Vaccinated | P |  | Unvaccinated | Vaccinated | P |
| Overall | 225/274(82.12) | 39/44(88.64) | 0.285 | Overall | 16.11(14.21,18.01) | 29.83(25.62,34.04) | <0.001^***^ |
| Sex |  |  |  | Sex |  |  |  |
| Male | 109/133(81.95) | 13/17(76.47) | 0.585 | Male | 15.06(12.44,17.69) | 33.04(26.66,39.43) | <0.001^***^ |
| Female | 116/141(82.27) | 26/27(96.30) | 0.065 | Female | 17.09(14.33,19.86) | 28.22(22.58,33.86) | <0.001^***^ |
| Age (ys) |  |  |  | Age (ys) |  |  |  |
| <20 | 14/17(82.35) | 0 | - | <20 | 12.67(4.61,20.72) | - | - |
| 20~ | 22/28(78.57) | 4/4(100.00) | 0.566 | 20~ | 15.46(8.36,22.55) | 21.16(-3.63,45.95) | 0.517 |
| 30~ | 50/64(78.13) | 12/15(80.00) | 0.874 | 30~ | 16.28(12.11,20.46) | 29.45(20.88,38.02) | 0.006^**^ |
| 40~ | 36/44(81.82) | 8/10(80.00) | 0.894 | 40~ | 17.56(13.05,22.08) | 26.88(14.35,39.41) | 0.088 |
| 50~ | 57/64(89.06) | 10/10(100.00) | 0.583 | 50~ | 16.33(12.40,20.26) | 34.88(27.79,41.96) | <0.001^***^ |
| ≥60 | 46/57(80.70) | 5/5(100.00) | 0.575 | ≥60 | 15.86(11.52,20.21) | 32.29(17.40,47.18) | 0.019^*^ |
| Severity of disease |  |  |  | Severity of disease |  |  |  |
| Asymtomatic type | 60/82(73.17) | 6/10(60.00) | 0.383 | Asymtomatic type | 12.96(9.79,16.12) | 28.98(13.16,44.79) | 0.004^**^ |
| Mild type | 42/46(91.30) | 13/14(92.86) | 0.854 | Mild type | 15.55(10.94,20.15) | 31.71(23.43,39.99) | <0.001^***^ |
| Normal type | 120/143(83.92) | 19/19(100.00) | 0.059 | Normal type | 17.75(15.00,20.49) | 28.35(22.28,34.43) | 0.005^**^ |
| Severe/Critical type | 3/3(100.00) | 1/1(100.00) | - | Severe/Critical type | 21.37(-21.34,64.08) | 38.46 | - |
| Months after symptom onset |  |  |  | Months after symptom onset |  |  |  |
| 15~16M | 150/174(86.21) | 36/41(87.80) | 0.788 | 15~16M | 16.86(14.48,19.23) | 28.95(24.50,33.39) | <0.001^***^ |
| 17~18M | 7/7(100.00) | 3/3(100.00) | - | 17~18M | 30.13(16.57,43.69) | 40.4(36.00,44.80) | 0.276 |
| *P* < 0.05 represents significant difference. *p<0.05; **p<0.01; ***p<0.001; ****p<0.0001 | | | | | | | |

**Table S13 Vaccination information for 44 recovered patients.**

| code | Sex | Age(ys) | Severity of disease | Sampling time | Date for dose 1 | Date for dose 2 | Interval between sampling  and last vaccination | Type of vaccine |
| --- | --- | --- | --- | --- | --- | --- | --- | --- |
| 1 | male | 32 | Normal type | 12-May-21 | 11-May-21 | - | 1 dose and < 14d | Sinovac |
| 2 | female | 24 | Normal type | 24-May-21 | 12-May-21 | - | 1 dose and < 14d | Sinovac |
| 3 | male | 36 | Asymtomatic type | 24-May-21 | 12-May-21 | - | 1 dose and < 14d | Sinovac |
| 4 | female | 38 | Asymtomatic type | 24-May-21 | 12-May-21 | - | 1 dose and < 14d | Sinovac |
| 5 | female | 43 | Normal type | 24-May-21 | 12-May-21 | - | 1 dose and < 14d | Sinovac |
| 6 | male | 44 | Mild type | 12-May-21 | 10-May-21 | - | 1 dose and < 14d | Sinovac |
| 7 | female | 73 | Asymtomatic type | 14-May-21 | 10-May-21 | - | 1 dose and < 14d | Sinovac |
| 8 | female | 49 | Normal type | 14-May-21 | 10-May-21 | - | 1 dose and < 14d | Sinovac |
| 9 | female | 48 | Mild type | 11-Jun-21 | 31-May-21 | - | 1 dose and < 14d | Sinovac |
| 10 | male | 48 | Asymtomatic type | 14-May-21 | 10-May-21 | - | 1 dose and < 14d | Sinovac |
| 11 | female | 31 | Asymtomatic type | 15-May-21 | 13-May-21 | - | 1 dose and < 14d | Sinovac |
| 12 | female | 22 | Mild type | 14-May-21 | 10-May-21 | - | 1 dose and < 14d | Sinovac |
| 13 | male | 43 | Normal type | 14-May-21 | 29-Mar-21 | - | 1 dose and ≥ 14d | Sinovac |
| 14 | male | 50 | Normal type | 14-May-21 | 19-Apr-21 | - | 1 dose and ≥ 14d | Sinovac |
| 15 | female | 21 | Normal type | 24-May-21 | 1-May-21 | - | 1 dose and ≥ 14d | Sinovac |
| 16 | male | 37 | Normal type | 16-Jun-21 | 29-Apr-21 | - | 1 dose and ≥ 14d | Sinovac |
| 17 | female | 30 | Mild type | 16-Jun-21 | 21-Apr-21 | - | 1 dose and ≥ 14d | Sinovac |
| 18 | male | 58 | Normal type | 13-May-21 | 16-Apr-21 | - | 1 dose and ≥ 14d | Sinovac |
| 19 | female | 47 | Mild type | 14-May-21 | 15-Apr-21 | - | 1 dose and ≥ 14d | Sinovac |
| 20 | female | 22 | Normal type | 13-May-21 | 22-Apr-21 | - | 1 dose and ≥ 14d | Sinovac |
| 21 | female | 37 | Mild type | 16-Jun-21 | 24-Apr-21 | - | 1 dose and ≥ 14d | Sinovac |
| 22 | male | 39 | Normal type | 12-May-21 | 24-Apr-21 | - | 1 dose and ≥ 14d | Sinovac |
| 23 | male | 31 | Asymtomatic type | 16-Jun-21 | 21-Apr-21 | - | 1 dose and ≥ 14d | Sinovac |
| 24 | male | 35 | Mild type | 24-May-21 | 26-Apr-21 | - | 1 dose and ≥ 14d | Sinovac |
| 25 | female | 43 | Mild type | 24-May-21 | 7-May-21 | - | 1 dose and ≥ 14d | Sinovac |
| 26 | male | 57 | Normal type | 14-May-21 | 15-Apr-21 | - | 1 dose and ≥ 14d | Sinovac |
| 27 | female | 64 | Normal type | 16-Jun-21 | 30-May-21 | - | 1 dose and ≥ 14d | Sinovac |
| 28 | female | 46 | Normal type | 13-May-21 | 1-Apr-21 | - | 1 dose and ≥ 14d | Sinovac |
| 29 | female | 56 | Asymtomatic type | 11-May-21 | 23-Mar-21 | - | 1 dose and ≥ 14d | Sinovac |
| 30 | male | 65 | Normal type | 24-May-21 | 1-May-21 | - | 1 dose and ≥ 14d | Sinovac |
| 31 | female | 60 | Mild type | 16-Jun-21 | 28-May-21 | - | 1 dose and ≥ 14d | Sinovac |
| 32 | male | 50 | Mild type | 12-May-21 | 3-Apr-21 | - | 1 dose and ≥ 14d | Sinovac |
| 33 | male | 58 | Normal type | 14-May-21 | 29-Apr-21 | - | 1 dose and ≥ 14d | Sinovac |
| 34 | female | 50 | Mild type | 10-Jun-21 | 22-May-21 | - | 1 dose and ≥ 14d | Sinovac |
| 35 | female | 34 | Asymtomatic type | 15-May-21 | 25-Apr-21 | - | 1 dose and ≥ 14d | Sinovac |
| 36 | female | 51 | Asymtomatic type | 12-May-21 | 28-Apr-21 | - | 1 dose and ≥ 14d | Sinovac |
| 37 | female | 37 | Mild type | 16-Jun-21 | 23-Apr-21 | 11-Jun-21 | 2 dose and < 14d | Sinovac |
| 38 | female | 32 | Mild type | 24-May-21 | 25-Mar-21 | 21-May-21 | 2 dose and < 14d | Sinovac |
| 39 | female | 63 | Normal type | 21-Jun-21 | 20-May-21 | 15-Jun-21 | 2 dose and < 14d | Sinovac |
| 40 | female | 32 | Severe/Critical type | 11-Jun-21 | 11-Apr-21 | 31-May-21 | 2 dose and < 14d | Sinovac |
| 41 | female | 51 | Normal type | 24-May-21 | 31-Mar-21 | 19-May-21 | 2 dose and < 14d | Sinovac |
| 42 | female | 39 | Asymtomatic type | 10-Jun-21 | 31-Mar-21 | 4-May-21 | 2 dose and ≥ 14d | Sinovac |
| 43 | male | 51 | Mild type | 24-May-21 | 25-Mar-21 | 27-Apr-21 | 2 dose and ≥ 14d | Sinovac |
| 44 | male | 49 | Normal type | 21-Jun-21 | 11-Apr-21 | 30-May-21 | 2 dose and ≥ 14d | Sinovac |
